# Lung Sonography in Critical Care Medicine

**DOI:** 10.3390/diagnostics12061405

**Published:** 2022-06-06

**Authors:** Robert Breitkopf, Benedikt Treml, Sasa Rajsic

**Affiliations:** Department of Anaesthesiology and Intensive Care Medicine, Medical University Innsbruck, 6020 Innsbruck, Austria; robert.breitkopf@tirol-kliniken.at (R.B.); benedikt.treml@tirol-kliniken.at (B.T.)

**Keywords:** lung sonography, COVID-19, critically ill, invasive procedure, intensive care, monitoring, point-of-care

## Abstract

During the last five decades, lung sonography has developed into a core competency of intensive care medicine. It is a highly accurate bedside tool, with clear diagnostic criteria for most causes of respiratory failure (pneumothorax, pulmonary edema, pneumonia, pulmonary embolism, chronic obstructive pulmonary disease, asthma, and pleural effusion). It helps in distinguishing a hypovolemic from a cardiogenic, obstructive, or distributive shock. In addition to diagnostics, it can also be used to guide ventilator settings, fluid administration, and even antimicrobial therapy, as well as to assess diaphragmatic function. Moreover, it provides risk-reducing guidance during invasive procedures, e.g., intubation, thoracocentesis, or percutaneous dilatational tracheostomy. The recent pandemic has further increased its scope of clinical applications in the management of COVID-19 patients, from their initial presentation at the emergency department, during their hospitalization, and after their discharge into the community. Despite its increasing use, a consensus on education, assessment of competencies, and certification is still missing. Deep learning and artificial intelligence are constantly developing in medical imaging, and contrast-enhanced ultrasound enables new diagnostic perspectives. This review summarizes the clinical aspects of lung sonography in intensive care medicine and provides an overview about current training modalities, diagnostic limitations, and future developments.

## 1. Introduction

Since it was first described back in the year 1968, lung sonography has now become an irreplaceable bedside tool in modern intensive care medicine. It can be used for the initial diagnostic assessment of a pulmonary pathology, periprocedural monitoring, or subsequent reevaluation of therapeutic interventions [1,2].

Lung sonography shows a high diagnostic accuracy for the most common etiologies of respiratory failure, such as pneumothorax, pulmonary edema, pneumonia, or pleural effusion. In critical care medicine, it has demonstrated better diagnostic sensitivity for respiratory pathologies than chest radiography [3,4,5,6,7,8,9]. Lung sonography correlates well with the severity of acute respiratory distress syndrome (ARDS) and may be used for mortality prediction in these patients. A special role of lung sonography is the guidance and monitoring of invasive procedures, fluid therapy, mechanical ventilation, respirator weaning, or antimicrobial therapy of critically ill patients [10,11,12,13,14].

In this review, we summarize and discuss the most current information about the clinical aspects of lung sonography in intensive care medicine, with a special focus on its use as a point-of-care diagnostic, including its advantages and limitations. Furthermore, we provide an overview of current training modalities and provide an outlook on future directions and perspectives.

## 2. Point-of-Care Ultrasound

The point-of-care approach combines the benefits of a fast, real-time, dynamic, cost-effective and non-invasive diagnostic evaluation with extremely high feasibility and patient safety, as the exposure to ionizing radiation or contrast dye is avoided [15,16,17]. Thus, lung sonography is particularly suitable for patients at risk, such as critically ill infants, children, or pregnant women [18,19]. 

Point-of-care ultrasound can be easily reproduced, requiring only basic, usually ubiquitous equipment, which is usually portable and lightweight, with built-in memory to store images and videos. Due to its acceptable cost, it is also accessible for low- and middle-income countries with limited healthcare resources [20]. 

Furthermore, the point-of-care sonography approach may reduce the likelihood of spreading hospital-acquired infections by avoiding intra-hospital transports of critically ill patients, minimizing the risk of additional healthcare staff exposure, while optimizing the availability of hospital resources and maintaining possibly necessary isolation measures for patients with infectious diseases [21,22]. This subject became especially important in the time of the coronavirus disease 2019 (COVID-19) pandemic, with intensive care units reaching their capacity limits, necessitating additional diagnostic methods for chest scans or radiography [23]. Thus, the constrained resources of stationary and constantly required imaging devices, such as CT scanners, can be retained for non-infectious patients, while the unused and mobile sonographic devices from departments that are operated less during the pandemic (e.g., outpatient clinics, specialized wards, etc.) can be made available to specialized COVID-19 units [24]. 

The immediacy of its results can be of extraordinary importance for the treatment of critically ill patients [25,26], enabling the examining clinician to directly correlate the findings of sonography with the potential cause of acute instability or condition deterioration [27].

## 3. Technique

Ultrasound is defined as a frequency of more than 20,000 Hz (20 kHz), exceeding the human audible range. For diagnostic ultrasound, frequencies in the millions of hertz (MHz) are used. The ultrasound waves are generated by applying electric current on piezoelectric crystals. When radiated into tissues, the ultrasound waves are transmitted, attenuated, absorbed, reflected, refracted, and diffracted by the difference in the acoustic impedance of various tissues [28]. 

Generally speaking, ultrasound waves penetrate well through fluid and solid organs, but are predominantly reflected by bone or air. This provides information regarding the location and characteristics of tissues. Lower frequencies (3–5 MHz) have a better penetration at the cost of a lower resolution, whereas higher frequencies (5–12 MHz) provide better images quality, but are limited in the visualization of deep structures [27]. 

Lung sonography is commonly performed using either B- or M-mode: B-mode, or brightness mode, is the basic mode of ultrasound imaging, generating the standard grey-scaled image on the ultrasound monitor. The M-mode, or motion mode, is used to visualize the movement of structures in a one-dimensional plane over time [29].

In critically ill patients, often intubated and ventilated, ultrasound investigations are usually performed in a supine position. In 2008, Lichtenstein et al. described the BLUE (bedside lung ultrasound in emergency) protocol, examining the lung by longitudinal scans at three sites: the upper anterior point, the lower anterior point, and the posterior lateral alveolar, or pleural point [30]. To be able to examine the latter site, the patient needs to be turned a bit to the contralateral side.

During the ongoing COVID-19 crisis, Soldati et al. developed a standardized sonography protocol, originally examining fourteen predefined areas. Each of them was further assigned a score from 0 to 3, based on the underlying pattern (normal = 0; “ground-glass” opacity = 1; interstitial changes = 2; consolidation = 3) [31]. Later, the lung ultrasound score (LUS) was calculated using a similar examination of twelve thoracic areas: anterior, lateral, and posterolateral views in the upper and lower thoracic walls on each side [10,32]. This semiquantitative assessment of pulmonary loss of aeration was then proposed to assess changes after various therapeutic interventions in mechanically ventilated patients [10,13,33]. 

## 4. Lung Sonography Signs, Patterns, and Clinical Interpretation

**Figure 1:**
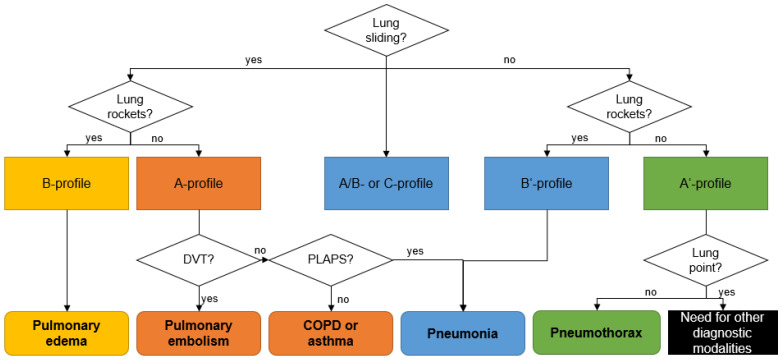
Sonographic decision tree according the BLUE-protocol. COPD: chronic obstructive pulmonary disease; PLAPS: posterolateral alveolar and/or pleural syndrome; DVT: deep venous thrombosis. Adapted from Lichtenstein et al. [30].

In the chest, aerated and liquid areas with different acoustic impedances border one another, creating ten typical sonographic signs (bat sign, lung sliding, A-lines, quad sign, sinusoid sign, squad sign, tissue-like sign, B-lines, stratosphere sign, and the lung point) of lung ultrasound. Among these, Lichtenstein et al. define eight sonographic patterns indicating essential respiratory diseases (Table 1 and Figure 1), with an overall accuracy of 90.5% [30].

**Figure 2:**
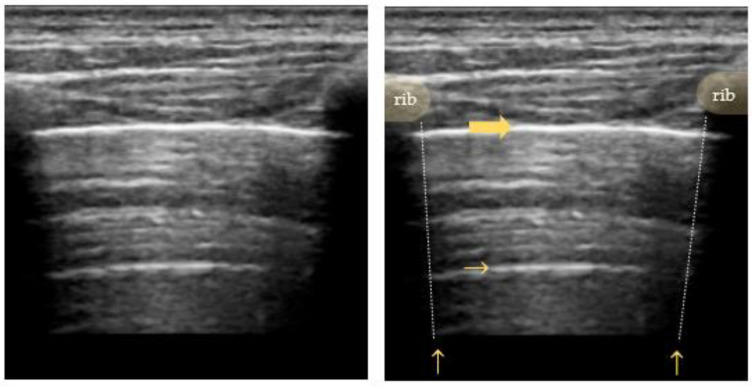
Bat sign: ribs and their sonographic shadow are outlined; the fat arrow points to the pleural line; the small horizontal arrow points to the A-line (reverberation artifact of the pleural line); the vertical arrow points to a rib’s shadow line.

In the following paragraphs, a short overview of typical sonographic signs is provided:

The pleural line is visualized as a horizontal hyperechoic line located in between the ribs. Its characteristic appearance in relation to the adjacent ribs is called the “**bat sign**”, with the ribs resembling the wings and the pleural line underneath representing the body of a bat. As the ultrasound waves are reflected by bony structures, the ribs cast a typical shadow sign. A high gas–volume ratio below the parietal pleura causes periodically recurring horizontal reverberation artifacts, so-called “**A-lines**” (Figure 2). They are repeated at a constant distance equal to the distance between the skin surface and the pleural line [34,35,36,37].

**Figure 3:**
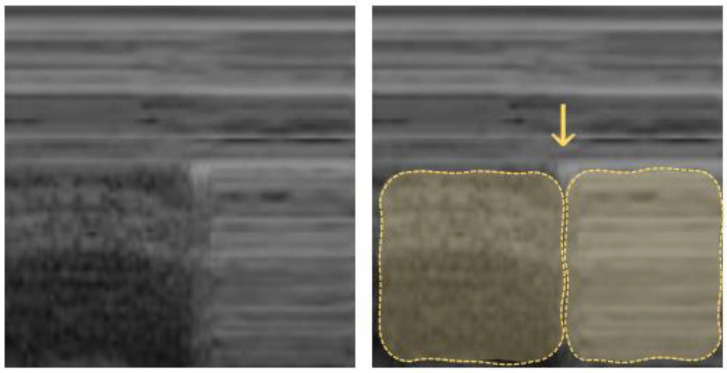
Pneumothorax (M-mode): “seashore sign” on the left (lung sliding of lung tissue creates a sand-like pattern) and “stratosphere sign” on the right (parallel lines created by abolished lung sliding); the vertical arrow points to the lung point in between.

Normally, the visceral and parietal pleura are closely associated, and ultrasound shows a periodical “**lung sliding**” movement at the pleural interface that is synchronous with the regional ventilation and can be further visualized by power Doppler (“power slide sign”). In the M-mode, lung sliding creates the characteristic “seashore sign” (Figure 3), where due to the motionless chest wall, the superficial tissues are represented as hyperechogenic horizontal lines, and the underlying tissue below moves away or toward the probe, creating a sandy pattern on the monitor [38,39].

A predominant A-line pattern, with preserved lung sliding and no pleural effusion, has been defined as a so-called “A-profile.” It has been shown to be associated with a pulmonary artery occlusion pressure < 18 mmHg. In patients with respiratory complaints, it may indicate a pulmonary embolism, especially if seen in combination with the verification of a deep venous thrombosis (A-DVT profile). Without deep venous thrombosis, it may indicate asthma or chronic obstructive pulmonary disease (COPD) [30,40]. 

**Figure 4:**
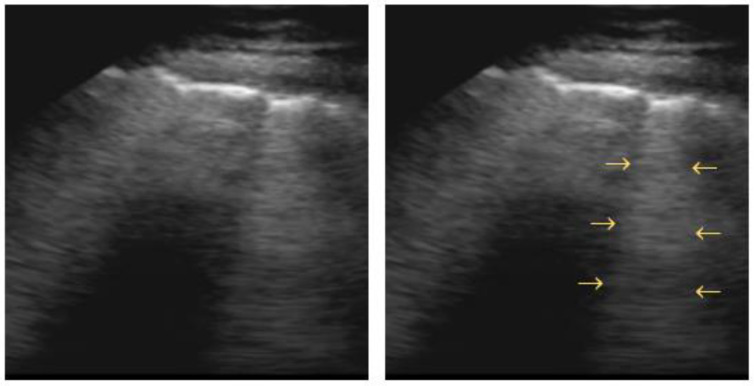
B-line: arrows point to vertical hyperechoic “comet-tail” artifacts arising from the pleural line and moving along with lung sliding.

“**B-lines**” are hyperechoic vertical artifacts resembling “comet tails” and moving synchronously with respiration. These B-lines arise from reflections created by a small air–fluid interface (Figure 4). They derive from the pleural line reaching the bottom of the screen, where they usually erase the A-lines [41].

Apart from these physiological ultrasound phenomena, pathological findings can be associated with specific etiologies of respiratory disease or failure. These were first summarized in the evidence-based recommendations of Volpicelli et al. in 2012 [42]. On the basis of 320 works published between 1966 and 2010, 28 experts elaborated 73 recommendations for the diagnosis of pneumothorax, interstitial syndrome, lung consolidation, and pleural effusion.

### 4.1. Pneumothorax

A pneumothorax is an abnormal collection of air in the pleural space between the lung and the chest wall leading to a partial or complete collapse of the underlying lung tissue. It can be caused by blunt or penetrating chest trauma, medical procedures, or damage from underlying lung disease and may lead to a life-threatening condition. According to the 2012 international consensus conference recommendations, the context of the following four sonographic signs define the diagnosis of a pneumothorax:

1. Absence of lung sliding;
2. Absence of B-lines;
3. Absence of lung pulse;
4. Presence of lung point(s) [42].

The absence of lung sliding may indicate a pneumothorax with context-dependent accuracy. It may be well visualized on M-mode images, as it appears in the form of uniform horizontal straight lines above and below the pleural line, known as the “**stratosphere sign**” (Figure 3) [43,44]. According to the BLUE-protocol, its combination with an anterior abolished lung sliding forms the so-called “A’-profile” [30]. However, the stratosphere sign is also seen in the case of lung overdistension in patients with COPD or artificial high positive end-expiratory pressure (PEEP) mechanical ventilation [45,46].

**Figure 5:**
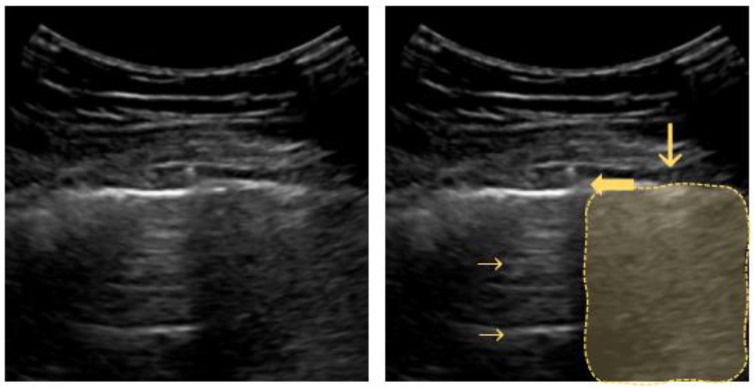
Pneumothorax (B-mode): the fat arrow points at the lung point; the small horizontal arrows point at A-lines; the vertical arrow points to the A’-profile (abolished lung sliding with exclusive A-lines).

Depending on the size of the pneumothorax, a “**lung point**” can be seen at the alternation of normal and abolished sliding during tidal ventilation in two-dimensional (2D) imaging (Figure 5), or visualized as a sharp transition from the seashore to the stratosphere sign in M-mode (Figure 3) [47].

Impaired regional ventilation, due to an airway obstruction, hyperinflation, pleural adherence, or bullae, also blur pleural sliding. However, a preserved contact of the visceral and parietal pleura creates a so-called “lung pulse” sign in M-Mode. These pulsations, synchronous with heartbeat but not with respiration, exclude a possible pneumothorax [43,48]. 

Lichtenstein et al. reported a sensitivity of 95.3% and a specificity of 91.1% for sonographic proof of pneumothorax [49], and Blaivas et al. demonstrated a sensitivity of 98.1% with a specificity of 99.2% in trauma patients [50]. In a study on extended focused assessment with sonography for trauma (EFAST), lung sonography was proven to be more than twice as sensitive for detecting pneumothorax, when compared to chest radiography, with a similarly high specificity (>98%) [51]. As long as there is no complete collapse of the lung, the presence of a lung point increases the specificity for the detection of pneumothorax to nearly 100% and has been shown to be a useful predictor of the pneumothorax volume [39,47,52]. Furthermore, if used on a routine basis, ultrasound can detect otherwise occult pneumothoraces and can be used to accurately assess pneumothorax progression during positive pressure ventilation [53,54]. However, small pneumothoraces may still be missed, and patients with altered lung parenchyma (as seen in the presence of bullae, contusions, or adhesions) may present false positive findings [55].

### 4.2. Interstitial Syndrome

Interstitial syndrome is an umbrella term for several pathologies exhibiting increased interstitial fluid, thereby reducing alveolar air, with at least partially preserved lung aeration [56]. Back in 1997, Lichtenstein et al. described for the first time the comet-tail artifact of B-lines as an ultrasound sign of an interstitial syndrome [41]. This comet-tail artifact can be correlated to several clinical conditions, such as pulmonary edema, ARDS, chronic interstitial diseases, as well as focally with infectious or ischemic processes [30,57,58,59]. For aortic stenosis, it was shown that the number of B-lines correlates with the hemodynamic changes caused by the valve lesion and the functional status of the patient [60].

The 2012 international consensus recommends the scanning of eight chest regions (upper and lower anterior, and upper and lower lateral on each side), which are then defined as positive by the presence of three or more B-lines in a longitudinal plane between two ribs. Finally, this indicates an impaired air–tissue ratio, with increased density [42,61]. 

While a focal B-pattern needs to be interpreted in the context of consolidations and pleural effusions, a diffuse B-line pattern, together with other sonography findings, may differentiate between [57]:

- Cardiogenic edema, with its homogeneous distribution of multiple diffuse bilateral B-lines, along with a regular thin pleura, normal sliding, and eventual bilateral pleural effusion (“B-profile”) [30,62];
- Pulmonary fibrosis, with B-lines in a diffuse, more or less homogeneous distribution, with an irregular pleural line and often, subpleural abnormalities [63];
- Acute respiratory distress syndrome, with a likewise nonhomogeneous B-line distribution with spared areas (“A/B-profile”), along with an irregular pleural line and reduced lung-sliding (“B’-profile”), or subpleural consolidations (“C-profile”) [30,62].

The sonographic technique for the diagnosis of interstitial syndrome has been shown to be superior to conventional chest X-ray [64]. The proof of B-lines excludes pneumothorax with a sensitivity of 100%, a specificity of 60%, and a negative predictive value of 100% [41]. Lung sonography allows for a bedside distinction between pulmonary edema and COPD, with a sensitivity of 100% and a specificity of 92% [65]. The described sonographic findings are present in 84.9–100% of cases in diffuse parenchymal lung diseases (fibrosis, sarcoidosis, silicosis, etc.), and the distribution of B-lines correlates with computed tomography (CT) signs of fibrosis [66]. 

Lung sonography can also contribute additional information to hemodynamic monitoring. In the fluid administration limited by lung sonography protocol (FALLS), the presence of an A-profile could be correlated with a pulmonary artery occlusion pressure equal to or lower than 18 mmHg (93% specificity and 97% positive predictive value). Together with an emergent cardiac sonography for diagnosing a tamponade or pulmonary embolism and a BLUE-protocol for the detection of pneumothorax (obstructive shock), and in combination with an investigation of the caval veins for exclusion of cardiogenic pulmonary edema, the FALLS protocol can be used to further differentiate two types of shock. On the one hand, an A-profile, with improvement after fluid administration, indicates a hypovolemic shock; on the other hand, a change from an A to a B-profile, with hemodynamic improvement after fluid administration, might indicate an overcorrection of the fluid deficit, whereas a change from an A to a B-profile, without hemodynamic improvement after fluid administration, suggests a distributive septic shock, with capillary leakage (Table 2) [40].

Copetti et al. showed that lung sonography is able to distinguish between a hemodynamic edema and pneumonia or ARDS. The first creates a B-profile, with conserved lung sliding due to transudation; the latter creates a profile of spared areas (A/B-profile), lung consolidations (C-profile), or pleural line modifications (C-profile) due to inflammatory exudation [62].

In the case of ARDS, lung sonography findings may predict mortality (area under the curve of 0.85), correlate well with the prognostic value of the invasively measured extravascular lung water index, and are able to pre-assess the post-extubation distress after a successful spontaneous breathing trial (area under curve of 0.86) [10,67]. Furthermore, point-of-care lung sonography helps monitor an early fluid loading in ARDS [13] and antibiotic-induced pulmonary reaeration in ventilator-associated pneumonia [68]. In ARDS patients supported with an extracorporeal membrane oxygenation (ECMO), lung sonography allows for the monitoring of the disease course and may indicate eventual lung recovery, with the potential of early ECMO weaning initiation and a consequent improved patient outcome [69,70]. 

Chiumello et al. found a strong association of ultrasound findings with the average lung density in CT scans, and Bouehemad et al. reported on a sonographic monitoring of the PEEP-induced lung reaeration. However, earlier research showed conflicting results: neither lung sonography was able to show effective pulmonary recruitability, nor to predict the oxygenation response to prone position ventilation due to its incapacity to detect hyperinflation [12,14,71,72]. Therefore, a supposedly pleasing reduction in global lung density can either correspond to a successful recruitment of atelectatic lung areas, or to an undesirable regional overdistension.

Alveolar overdistension can lead to volutrauma with disruption of the alveolar capillary membrane, a consecutive overactivation of the pulmonary inflammatory response to biotrauma causing further edema [73].

### 4.3. Lung Consolidation

Lung consolidations represent a lung area in which alveolar air is replaced by exudate or other secretions, rendering the lung solid [74]. They show a high variability in their depiction and can be further specified in the context of additional sonographic signs, such as the air bronchogram(s), the quality of its margins, the B-lines, or the vascular pattern within the consolidation. 

Consolidations may be a result of different pathological processes, for example, pneumonia, compression or obstructive atelectasis, lung contusion, pulmonary embolism, pleural disease, or neoplasia [43,48,66,75]. 

Pneumonia progresses though stages: in the beginning, fluid-filled alveoli are surrounded by the air-filled lung, which is sonographically visualized as the previously described B’-profile, with its anterior diffuse B-lines and abolished lung sliding. The changes may be widespread, but also patchy or lobar, which is then seen as the A/B-profile under ultrasound. As soon as inflammatory and purulent fluid fills the alveoli, consolidations occur, creating the sonographic C-profile. They appear solid, with a homogenous echotexture, and unchanged or eventually increased lung volume. Small, ventilated lung spots are referred to as sonographic air bronchograms; the vascular system can be visualized sonographically in solidified areas, and accompanying pleural effusions (A-V-PLAPS-profile) may also occur [30,76]. 

**Figure 6:**
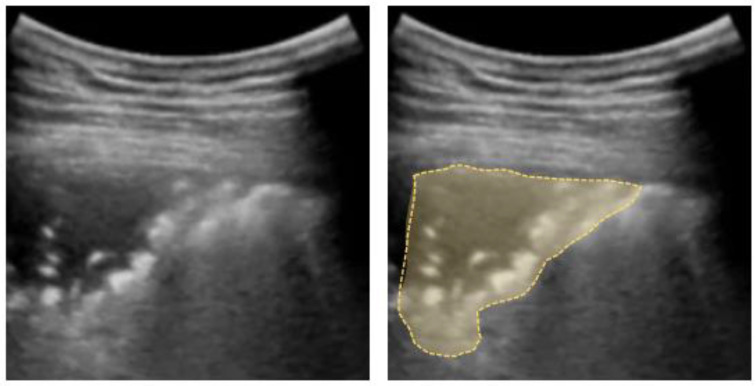
Shred sign: small nontranslobar echo-poor consolidation delimited by irregular boundaries (outlined).

The visualization of a small subpleural, echo-poor consolidation with irregular boundaries is called the “**shred sign**” (Figure 6). It is often associated with diffuse parenchymal lung diseases (e.g., fibrosis, sarcoidosis, interstitial pneumonia, silicosis, etc. [63]), but may also correspond to pulmonary subpleural infarcts after pulmonary embolism [77].

A completely consolidated lung, as seen in (accidental) one-lung ventilation, appears as a homogeneous gray “**tissue-like**” structure. This corresponds to complete air reabsorption and potentially not-patent airways [78].

Air bronchograms are visualized as hyperechoic signs within the consolidation and may provide additional information on the consolidation etiology. If static, it may be suspicious for an airway obstruction with incomplete air reabsorption, which is seen in 40–90% of pneumonias [76]. 

A dynamic air bronchogram, seen as multiple white spots moving synchronously with tidal ventilation, corresponds to patent airways and rules out obstructive atelectasis [76,78]. It is indicative of community-acquired pneumonia (sensitivity of 93.4% and specificity of 97.7%), with accuracy at least comparable to chest radiography results, with a capacity for increased diagnostic accuracy when combined with lung auscultation. About 8% of pneumonic lesions may be missed by ultrasound; consequently, an inconspicuous sonography does not completely exclude pneumonia [79,80]. 

A dynamic linear/arborescent air bronchogram is characteristic of a ventilator-associated pneumonia, with a positive predictive value of 86% and a positive likelihood ratio of 2.8. In case of visualization of two dynamic linear/arborescent air bronchograms, the positive predictive value increases up to 94%, with a positive likelihood ratio of 7.1 [81].

When combined with history and examination, lung sonography is capable of differentiating, with high accuracy, pneumonic consolidation from atelectasis and its numerous causes.

### 4.4. Pleural Effusion

Pleural effusions are the build-up of excess fluid between the layers of the pleura. They are a common finding in critically ill patients and are mainly caused by volume overload, congestive heart failure, pleuro-pulmonary infections, atelectasis, and cardiothoracic or major abdominal surgery. They affect ventilation by thoracic over-distension and impair oxygenation due to increasing shunt volume [82]. In addition to its value in diagnosing the presence of a pleural effusions, lung sonography allows for the evaluation of the echogenicity of the fluid and the presence and degree of septations, which is of great importance for performing further therapeutic procedures.

**Figure 7:**
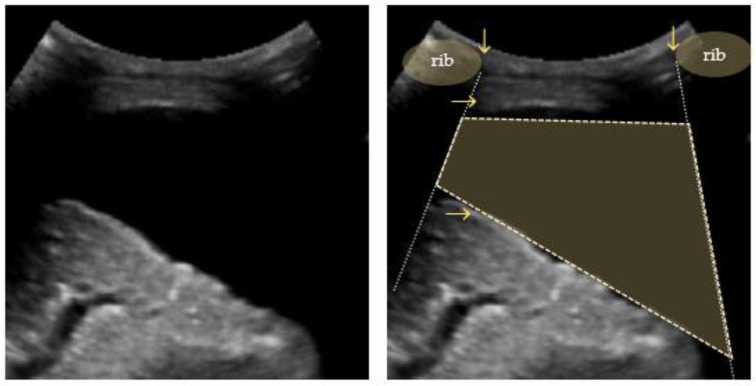
Quad sign: trapezium shaped pleural effusion (outlined) defined by the pleural and lung lines (horizontal arrows) and the shadows from the adjacent ribs (vertical arrows).

Pleural effusions are visualized as a hypoechoic space between the parietal and visceral pleura, with a freely floating lung within. This phenomenon can be described as a “**sinusoid sign**” in M-mode scans [83,84]. The visceral pleura line (lung line), together with the almost parallel parietal pleura (pleural line) and the shadows from the adjacent ribs, form a four-sided figure known as the “**quad sign**” (Figure 7) [34]. 

The measurement of the interpleural distance allows for the quantification of a pleural effusion [85,86,87]. The most accurate equation in predicting the effusion volume was published by Gecke and Schwerk in 1990: the maximal craniocaudal distance of effusion in centimeters is added to the height of the subpulmonal effusion and multiplied by the factor 70, resulting in the anticipated volume of the pleural effusion in milliliters [88]. In 1994, Eibenberger et al. proved that the detection of pleural effusion by sonography provides a higher sensitivity than either clinical examination or chest radiography [89].

The diagnostic accuracy for pleural effusions using lung sonography (97%) has been shown to be superior in comparison to auscultation (61%) and bedside chest radiography (47%) [90].

The combination of pleural effusions with an A-profile and the absence of deep venous thrombosis is a so called “A-V-PLAPS profile,” associated with pneumonia [30]. Depending on its echogenicity and the presence of septations, a pleural effusion can be further differentiated as transudate or exudate [83,91,92]. Finally, the presence of pleural or diaphragmatic thickening, nodularity, or an echogenic swirling pattern, together with a positive clinical anamnesis, may be suggestive of a malignant pleural effusion [93,94,95].

### 4.5. Diaphragmatic Dysfunction

A newer application of lung sonography is the functional evaluation of the primary muscle of respiration—the diaphragm. Diaphragmatic dysfunction is increasingly recognized as an important element, not only in critically ill patients, but also in several different diseases, including neuromuscular disease or chronic obstructive pulmonary disease. Diaphragm dysfunction can be classified as weakness, paralysis, and eventration [96]. Lung sonography enables both a static and dynamic evaluation to assess the integrity, excursion, thickness, and thickening of the diaphragm [97,98]. 

The diaphragmatic excursion is examined by a 3.5–5 MHz phased array probe placed immediately below the right or left costal margin in the mid-clavicular line, or in the right or left anterior axillary line, being directed medially, cephalad, and dorsally. In the M-mode, the diaphragmatic excursion (displacement, cm), the speed of diaphragmatic contraction (slope, cm/s), the inspiratory time (Tinsp, s) and the duration of the cycle (Ttot, s) can be measured. Diaphragmatic thickening and the thickening fraction can be examined by a linear high-frequency probe (≥10 MHz) in M-mode or 2D-mode. Pleural effusions, consolidation, or atelectasis generally allow an easier visualization [99].

Diaphragmatic dysfunction is usually diagnosed by an excursion depth less than 10 mm and may be associated with adverse outcomes in terms of duration of ventilation and intensive care unit (ICU) stay, as well as hospital mortality [99,100]. Diaphragm thickening fraction can be used as predictor for weaning failure with comparable accuracy to the rapid shallow breathing index (with a sensitivity of 82%, specificity of 88%, positive predictive value of 0.92, and negative predictive value of 0.75) [101]. Furthermore, it correlates with diaphragm and esophageal pressure-time product and may be used as a surrogate for the work of breathing [102,103].

Sonography has been shown to be superior to fluoroscopy as a diaphragmatic dysfunction diagnostic, a well described phenomenon among critically ill patients with ICU acquired weakness [104,105,106]. Although the thickening fraction could not be correlated with the Medical Research Council score, a score for grading muscular strength [107], ultrasonic assessed excursion or thickening can be used as a predictor of successful weaning from mechanical ventilation [99,108]. In this regard, a sonographic quantification of liver/spleen displacement has also been described as an alternative to direct examination of the diaphragm (sensitivity 84.4%, specificity 82.6%) [109].

## 5. Periprocedural Monitoring

Besides its well-known point-of-care use as guidance for the placement of central venous catheters [110], there are further possible indications for periprocedural ultrasound monitoring of invasive interventions in the care of the critically ill (e.g., tracheal sonography as confirmation for proper endotracheal tube position, guidance for pleural effusion drainage, pneumothorax puncture, etc.) [111,112,113,114,115]. 

Point-of-care ultrasound can further reduce the incidence of complications during thoracocentesis. Cavanna et al. described a significant reduction in pneumothorax incidence with the use of lung sonography, from 3 (0.97%) versus 12 (8.89%), without guidance. Furthermore, Gordon et al. and Mercaldi et al. confirmed a significantly lower risk of complications, with an odds ratio of 0.3 and 0.81, respectively. Diacon et al. showed that a sonographic identification of a proper puncture site may prevent a possible accidental organ puncture in 10% of all cases and increases the rate of successful puncture by 26% [84,116,117,118,119,120,121]. Lichtenstein et al. recommended a pleural fluid depth of at least 15 mm, visualized over three intercostal spaces, in mechanically ventilated patients to minimize the potential risk of complications [84]. Finally, potential postinterventional pneumothorax can be successfully excluded by point-of-care sonography [122].

Besides thoracocentesis, lung sonography can further reduce the hemorrhage risk of percutaneous dilatational tracheostomy by prior identification of pretracheal vascular structures. Moreover, ultrasound may guide the retraction of the endotracheal tube (ETT) to prevent cuff damage and ETT puncture when accessing the trachea with a needle, resulting in shorter surgery time when compared to a bronchoscopic guidance [123,124,125,126,127].

## 6. The Coronavirus Disease 2019 (COVID-19)

The COVID-19 pandemic has led to a veritable explosion in the use of lung sonography. 

As early as 2020, the typical sonographic signs of a COVID-19 infection, with its ARDS-like bilateral patchy distribution of multiform clusters, coalescent B-lines, small peripheral consolidations, and irregular, fragmented pleural line, besides spared areas, were described for the first time and correlated with CT findings [128,129,130,131]. Volpicelli et al. described the “light beam” (also known as the “waterfall sign”) in the early phase of COVID-19 pneumonia: a shining band-form artifact spreading down from a large portion of a regular pleural line, often appearing and disappearing with an on–off effect in the context of a normal A-line lung pattern visible on the background [131,132]. 

The Lung Ultrasound Score grading, based on the publication by Soldati et al., attributes a score of 0–3 to each of 12 standard defined regions which can then be used to quantify the loss of aeration and monitor reaeration over time. It was proven to be a good predictor of death, ICU admission, and endotracheal intubation in patients with COVID-19 [31]. 

Especially in the beginning of the pandemic, the bedside approach by a single operator was its greatest advantage, since it reduced the risk of cross contamination, as well as the healthcare workers’ viral exposure, and mitigated the initial shortages of personal protective equipment [132]. 

Boero et al. summarized the role of lung sonography in the management of COVID-19 patients from their initial presentation in the emergency department, during their hospitalization, and after their discharge into the community [133]. It can be helpful to correctly allocate patients to the right hospitals and to separate those needing admission from those who can be discharged safely. Its routine use prevents asymptomatic patients from being overlooked. On COVID-19 wards, it can be used to early recognize a deterioration, and in the ICU, to adjust and monitor the setting of and weaning from ventilation.

Recently, Kirkpatrick et al. examined the feasibility of tele-mentored self-performed pleural ultrasound assessment for the home surveillance of patients at risk for COVID-19 deterioration [134].

The potential for further utility of lung ultrasound in COVID-19 patients is still subject of extensive debate, but due to limited resources, personal and protective equipment shortage, and danger of infection spreading, the field of possible applications has already been greatly expanded. However, the role of ultrasound in the evaluation of COVID-19 has not yet been established, since it cannot detect pulmonary ground-glass opacification in the lungs, this being one of the cardinal CT scan findings of COVID-19 using standard imaging modalities. Besides, lung ultrasound also requires close contact between the patient and the examiner, imparting a potential risk for infection transmission. 

## 7. Training, Competence, and Credentialing

Lung ultrasound examination and correct interpretation of the resulting images requires formal training to acquire the necessary skills and certifications. 

Critical care sonography—covering lung, abdominal, and vascular sonography—was identified for the first time as a core competency in intensive care medicine and as mandatory in the intensivists curriculum after a roundtable discussion during the 23rd annual meeting of the European Society of Intensive Care Medicine (ESICM) in Vienna, October 2009. In the same year, an initial statement defining essential skills for an intensivist was published by the American College of Chest Physicians and La Société de Réanimation de Langue Française [135]. 

Despite precisely defined training standards on critical care sonography, most fellowship trained intensivists typically have their competence documented by their program director, without board certification [136]. In this context, recent surveys among critical care program directors and fellows have shown significant deficiencies in their practical implementation in critical care training [137,138]. Pietersen et al. systematized published evidence about lung ultrasound education and certification and concluded that due to missing research on competency-based training programs and assessment tools, it is not possible to construct clear training guidelines for the future [139].

Lung sonography can be seen as a technique which is easy to learn and simple to perform [33]. Lichtenstein et al. demonstrated a high accuracy of 93% for pleural effusion, 97% for alveolar consolidation, and 95% for alveolar-interstitial syndrome with minimal intra- and inter-observer variability, after only two months of training [90]. Several studies have confirmed that sonography skills can be satisfactorily attained after a training time of up to four months and with up to 100 supervised scans [59,80,140,141]. In a study on the assessment of competency in thoracic sonography, Millington et al. concluded a rapid early improvement in learner performance during their first 25 to 30 practice studies [142]. Depending on the knowledge and skills of the examiner, the duration of a complete examination may take about 15 min [72].

## 8. Limitations of Lung Sonography

Nevertheless, point-of-care lung sonography has its limitations. Depending on operator skills, visualization of lung parenchyma might be difficult in obese, postoperative, or trauma patients with subcutaneous emphysema or large thoracic dressings. Moreover, dorsal lung segments of the upper lobes are the blind spots, since they are located behind the scapula and cannot be explored by lung ultrasound [72].

Ultrasound probes and coupling gel, if used in several patients and without previous disinfection, might be a source of infection spreading. This is of special interest in the intensive care unit setting, where using single-patient gel, as well as probe and echo shields, including specific decontamination procedures, is recommended [143,144].

## 9. Future Perspectives

Deep learning is rapidly progressing in medical imaging. It is used for technical optimization of ultrasound image formation [145] and for automatic image analysis in machine and deep learning methods. In case of the COVID-19 infection, machine learning has been successfully used to assist clinicians in detecting COVID-19-associated imaging patterns on point-of-care lung sonography, with the possibility of simultaneous disease severity score prediction [146]. This kind of artificial intelligence is capable of automatically detecting, localizing, and classifying B-lines in an ultrasound scan [147,148]. 

Moreover, artificial intelligence is about to automatically assist in capturing images according to predefined protocols during the acquisition process, with real-time feedback which may help in the generation of standardized datasets which can be used for further development and training [149].

Finally, another important and developing field of sonographic research is the use of contrast-enhanced ultrasound. It is especially useful in the characterization of the perfusion level of lung consolidations [150]. 

Pneumonia, atelectasis, and embolic consolidations each show different patterns of parenchymal enhancement; while a pulmonary infection shows an inflammatory hyperperfusion, airway obstruction shows a homogeneous enhancement of tissue-like parenchyma, which might be completely missing or inhomogeneous in embolic consolidations. Long-lasting atelectasis or pneumonia might evolve into a more patchy perfuse inhomogeneity over the time, due to hypoxic vasoconstriction [151,152,153].

Several studies described atypical large perfusion defects, delimited with respect to the perfused parenchyma, which often appeared only as a shell of enhanced tissue in COVID-19 patients. This raised the question of whether the observed consolidations do not, in fact, represent atelectasis or easily recruitable areas, but tissue with large perfusion defects [153,154].

Lung sonography remains an emerging field of interest regarding pulmonary diseases and critical care medicine. Many authors have already demanded the need for further research to evaluate its diagnostic and prognostic value and to establish widely recognized standards.

## 10. Conclusions

Point-of-care lung sonography is a simple and easy-to-learn bedside tool with a wide range of possible clinical applications. Besides accurate diagnosis of the most common etiologies of respiratory disorders, it can also be used for the periprocedural monitoring of invasive interventions in the care of the critically ill. Moreover, aeration in mechanically ventilated patients can be monitored, with possible consequences for ventilator adaptation, prone positioning, respirator weaning, or fluid-therapy. The recent COVID-19 crisis has further increased its scope of clinical impact and proven its significance as a core point-of-care competence for modern intensive care medicine. Continuing education and dly implemented and offered, encouraging intensivists to expand their knowledge on sonography and stay up-to-date with the newest developments. While point-of-care lung sonography may not necessarily replace other diagnostic modalities, it is becoming an important and indispensable tool for improving the care and outcomes of critically ill patients.

## Figures and Tables

**Table 1:** The eight profiles of the BLUE protocol and their clinical interpretation [30].

BLUE Protocol Profile	Profile Description	Etiology of Respiratory Failure
A-profile	*Anterior lung sliding + A-lines + free veins*	Exacerbated COPD or Severe acute asthma
B-profile	*Anterior lung sliding + lung-rockets*	Pulmonary edema
B’-profile A/B-profile C-profile A-V-PLAPS profile	*B-profile + abolished lung sliding * *Half A-profile at one lung, half B-profile at another * *Anterior lung consolidation * *A-profile + free veins + PLAPS*	Pneumonia
A-DVT profile	*A-profile + DVT*	Pulmonary embolism
A’-profile	*A-profile + abolished lung sliding (+ lung point)*	Pneumothorax

BLUE: bedside lung ultrasound in emergency; COPD: chronic obstructive pulmonary disease; PLAPS: posterolateral alveolar and/or pleural syndrome; DVT: deep venous thrombosis.

**Table 2:** Sequential differentiation of shock by sonography according to the FALLS-protocol [43,73].

Sonographic Signs	Weil/Shubin Shock Classification	
Cardiac sonography	Tamponade, pulmonary embolism	Obstructive shock
BLUE: A’-profile	Pneumothorax	
BLUE: B-profile	Pulmonary edema	Cardiogenic shock
FALLS-protocol	Unchanged A-profile	Hypovolemic shock,
	Change from A- to B-profile	Septic shock

BLUE: Bedside Lung Ultrasound in Emergency; FALLS: Fluid Administration Limited by Lung Sonography.

## Data Availability

Not applicable.
